# The Africa Centres for Disease Control public health emergency management fellowship: insights from the inaugural cohort

**DOI:** 10.3389/fpubh.2025.1444354

**Published:** 2025-09-04

**Authors:** Mesfin Wossen Getaneh, Albert Pascal, Rejoice Kudirat Luka-Lawal, Martin Kukenzikila Kutubana, Joseph Anderson Bunting-Graden, Esmeraldo Tiago Camboneia Ezembro, Soransora D. Tadicha, Paulin N. Essone, Simon Magodi, Uchenna Anebonam, Wessam Mankoula, Womi Eteng Oboma

**Affiliations:** ^1^Africa Centres for Disease Control and Prevention, Addis Ababa, Ethiopia; ^2^Ethiopia Public Health Institute, Addis Ababa, Ethiopia; ^3^Public Health Emergency Operation Center, Ministry of Health, Kigoma, Tanzania; ^4^Nigeria Centre for Disease Control and Prevention, Federal Capital Territory, Abuja, Nigeria; ^5^Veterinary Services, Ministry of Fisheries and Livestock, Kinshasa, Democratic Republic of Congo; ^6^Health Security and Emergencies, Ministry of Health, Freetown, Sierra Leone; ^7^Department of Surveilance, Ministry of Health, Maputo, Mozambique; ^8^Public Health Emergency Operation Center, Isiolo, Kenya; ^9^National Laboratory of Public Health, Ministry of Health, Libreville, Gabon

**Keywords:** public health emergency management, public health emergency operations centre, workforce, fellowship, health security, emergency preparedness and response

## Abstract

Public health emergencies remain a significant challenge across the African continent, driven largely by the high burden of infectious diseases and constraints in the public health workforce. The Africa Centre for Disease Control and Prevention (Africa CDC) has taken a strategic move towards addressing emergency preparedness and response workforce gap with the implementation of the Public Health Emergency Essone Management (PHEM) Fellowship aimed at providing African Union Member States (MS) with trained personnel in emergency management. We describe the components of this program comprised of six sections such as the advisory committee (from several countries and divers organizations), a rigorous selection process, eleven weeks of in-person classes with validated materials, field deployments in countries with ongoing emergency crises, projects and mentorship programs. The PHEM fellowship offers African countries a strategic opportunity to strengthen their public health workforce and enhance their preparedness towards potential public health emergencies.

## Introduction

Public health emergencies in Africa present complex and multifaceted challenges. The current landscape reveals both progress and limitations specifically in Africa’s health emergency workforce capacity (1–3). According to the 2016 states parties self-assessment reports, no country in the African region had all the required International Health Regulation capacities (1, 2). Even though it bears 25% of the global burden of disease, it has only 3% of the world’s health workforce (3, 4). Africa Centre for Diseases Control (AfricaCDC) is a continental autonomous health agency of the African Union established with vision of a safer, healthier, and more prosperous Africa where Member States are prepared to timely prevent, detect, and respond effectively to public health threats (5–7). AfricaCDC, with its partners, have been making efforts to enhance workforce development by establishing strategies and programs to minimize the dire needs of workforce development, such as the Africa Volunteer Health Corps (AVoHC) (5, 6, 8).

The Public Health Emergency Management (PHEM) Fellowship was launched on March 16, 2023. The fellowship is envisioned as a critical source of skilled mid-career workforce for setting up and managing emergency preparedness and response programs, with particular focus on Public Health Emergency Operations Center (PHEOC) management. It represents a significant milestone in actualizing the 2022 Lusaka Call-to-Action on strengthening PHEOCs in Africa (9). The Call, made by heads of states and governments of the African Union, urged all stakeholders to deepen investments in PHEOCs as a critical component of the health security discourse. This report aims to highlight the various components of this initiative.

## Methodology

This is a descriptive study highlighting the AfricaCDC PHEM fellowship, focusing on insights drawn from the inaugural cohort. Data collection for this work was organized in several mini seminars at the end of the fellowship. All PHEM fellows participated in these semi seminars and revised all sections of the fellowship program. Two to three sections of the PHEM program were discussed during each mini seminar. Two fellows were designated to lead this work and initiate the manuscript writing. Each section of the program was discussed in detail and fellows were invited to give their personal experience and comments on the specific sections separately. At the end of these mini seminars, the two leaders gathered all inputs and initiated the report. The report included describing the main sections of the program including the advisory committee, the selection process, in-person classes, study tour, deployment and the post training project.

## Results

The 2023 AfricaCDC PHEM fellowship has been a successful experience where African mid-career health workforce were equipped to face and respond to public health challenges in their respective countries. This success could be attributed to the five sections briefly discussed here.

### Advisory committee

Africa CDC established an advisory group committee board for the PHEM fellowship. This advisory board was constituted with experienced public health emergency management experts from around the world including WHO, United Kingdom Health Security Agency (UKHSA), European Centre for Disease Control and Prevention (ECDC), Bill & Melinda Gates Foundation (BMGF), The Robert Koch Institute (RKI), Resolve to Save Life (RTSL) and United States Centers for Disease Control and Prevention (USCDC). This advisory committee supported the implementation of the PHEM program. They provided technical guidance for the overall implementation of the PHEM fellowship program. They had several online and in-person meetings to prepare this program. The committee revised and validated all steps and course materials before the start of the program.

### Selection process

**Figure 1:**
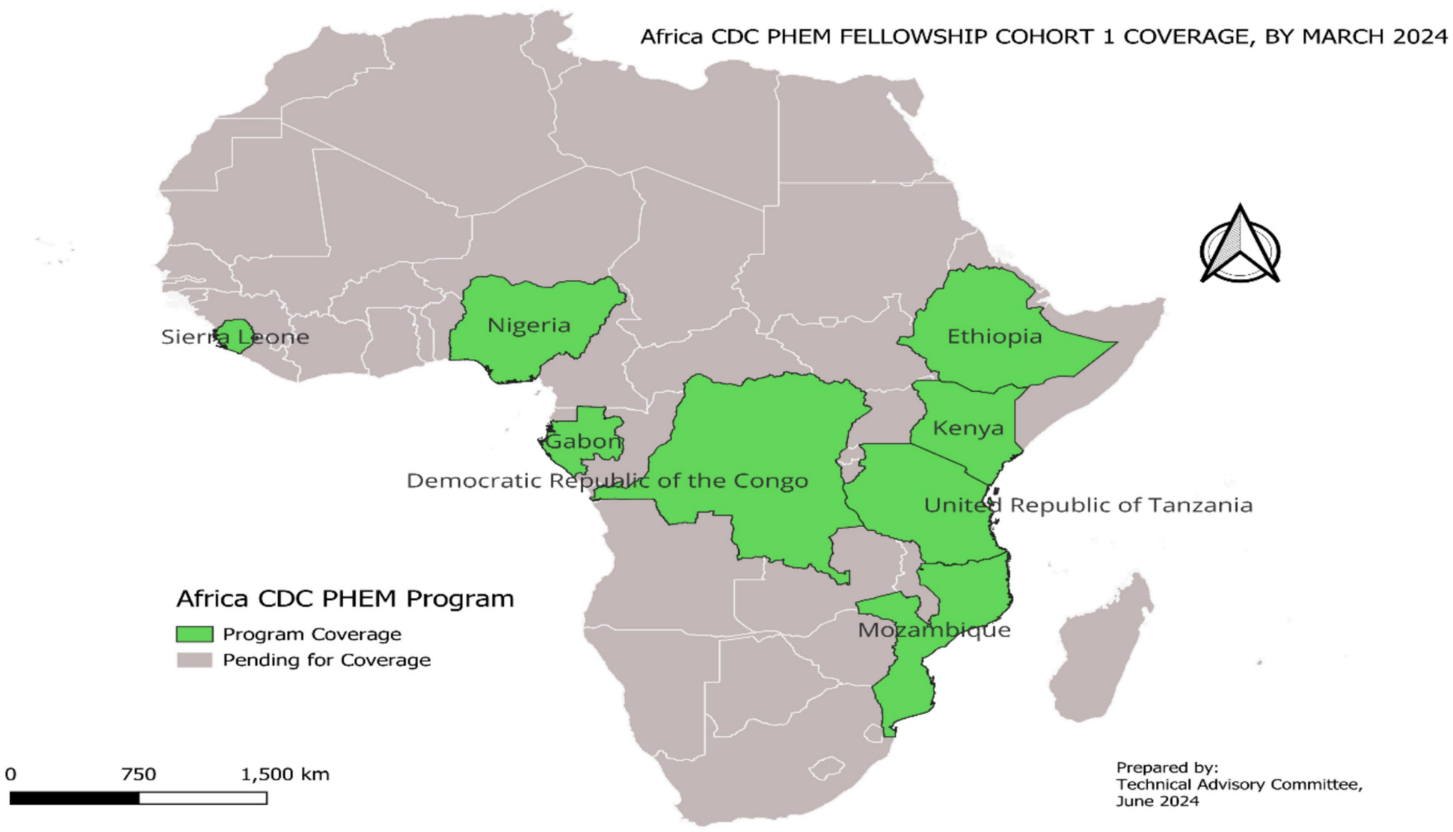
First cohort of AfricaCDC PHEM fellowship program coverage.

The end of validation process was followed by program announcement. The call was opened to all African Union Member States. More than 1700 applications were received during this call. After a careful screening and interview process, eight candidates were chosen from the Democratic Republic of the Congo (DRC), Ethiopia, Gabon, Kenya, Mozambique, Nigeria, Sierra Leone, and Tanzania. The selected candidates were from different parts of Sub-Saharan Africa as shown on Figure 1.

### In-person classes

The PHEM fellows were first enriched with in-person lectures, seminars and experience sharing. The first part of the fellowship program consisted of eleven (11) weeks of in-person classes divided in three (3) components: emergency and disaster management, public health science, and leadership and management (Table 1). On the first competency domain of the program (emergency and disaster management), fellows were exposed to the basic concepts of risk assessment, all-hazards emergency management, crisis communication, components of PHEOC, and incident management system (IMS). It also explored the four phases of emergency management: prevention/mitigation, preparedness, response, and recovery, and provided an overview of global health security regulations such as international health regulations and the pandemic accord. The second domain of competency (public health science) focused on concepts and methods of epidemiology, public health surveillance, and basic concepts of public health research, including quantitative and qualitative approaches which are essential tools for public health practice. At the end of the competency, fellows demonstrated improved knowledge on the basic principles and techniques of data collection, management, analysis, and interpretation of surveillance data, communicating and disseminating surveillance findings, and were familiarized with regional and global detection and reporting systems. The last competency of the fellowship program was leadership and management in health emergencies, in which fellows were introduced to the concepts and skills of leadership and management. Fellows practiced skills on how to influence people to achieve common goals using the tools and techniques of effective management practice. Additionally, fellows were exposed to several PHEM initiatives through didactic seminars such as The African Health Volunteers Corps (AVoHC), event based surveillance system, as well as AfricaCDC regular PHEOC and incident management system meetings.

**Table 1:** Fellowship competencies and courses.

S. N	Domain	Course	Modality
1.	Emergency and disaster management	Principles of emergency management	Lectures, group work
		Public health emergency preparedness	Lecture, group work and shadowing to AfricaCDC PHEOC
		PHEOC operations and management	Lecture, group work, shadowing to AfricaCDC PHEOC, study tour to Zambia NPHI and ECDC PHEOC
		Emergency response operations	Lecture, group work, shadowing to AfricaCDC PHEOC, study tour to Zambia NPHI and ECDC PHEOC
		Ethics and legal dimension of emergency management	Lectures, group work and case study
2.	Public health science	Epidemiology and surveillance	Lecture, group work, shadowing to AfricaCDC PHEOCs
		Public health research	Lecture, group assignment presentation
		Geographic information system	Lecture, group work and case study
3.	Leadership and management	Leadership	Lectures, group work
		Management	Lectures, group work

### Study tours

The fellows were invited to experience real time field activities in different countries with ongoing health emergency and to visit Emergency Operations Centers (EOC) in different countries and settings (study tours). A study tour was a short deployment from two to five days or more to an EOC where fellows were exposed to routine activities taking place in these centers. Several discussions with different experts were organized on selected days as part of the activities. Two study tours, in Zambia and Europe (Sweden and Lithuania), were organized. The study tour to Zambia gave Fellows the opportunity to visit the regional and national health security infrastructures established at the Africa CDC Southern Africa Regional Coordination Center (SA-RCC) and the Zambia National Public Health Institute. Fellows also used the opportunity to attend the Conference of Public Health in Africa (CPHIA 2023) with preselected sessions on the latest public health emergency response activities across the Africa. During the second study tour fellows visited the European Centre for Disease Control (ECDC) and the ministry of health of Lithuania. The tour included a short trip to the Lithuanian EOC center in Vilnius. Fellows gained better understanding on how public health emergency response is designed and supported at the European Union level. This included topics on preparedness plans, response activities, PHEOC, and the European Union’s emergency decision-making process. ECDC invited partners such as European Union Member States, the European Union Commission, and WHO-European Region to share experience with the fellows. Fellows captured experiences about how ECDC manages threat detection and epidemic intelligence and discussed the challenges and lessons learned from the COVID-19 crisis.

### Field deployments

The fellowship program was enriched by field deployment experience for twenty-one days per deployment. Fellows were divided into three groups. Two groups were, respectively, deployed for management of cholera outbreak in Zambia and Zimbabwe. The third group was deployed for PHEM system strengthening in Libya following a flood disaster in late 2023. The deployment afforded fellows a practical hands-on demonstration of knowledge acquired in the classroom sessions. During the deployment periods, fellows were able to support ongoing outbreak response by preparing incident action plans (IAPs), situation reports, supervision and training of community health workers recruited by AfricaCDC under the supervision of a mentor.

### Post training projects

Part of the entry requirements for the fellowship was the submission of a proposal addressing a major public health challenge in the fellow’s home country. As a post-training fulfilment requirement, Fellows were expected to develop business cases for implementation of the identified projects when they return to their home countries. The DRC had declared several outbreaks of zoonotic origin in recent years, but the flow of information from peripheral to central levels is still challenging. The program benefited from the participation of one expert from the DRC with a strong background in veterinary medicine and experience in surveillance for zoonotic diseases. Taking advantage of the current fellowship and knowledge gained, a protocol was established to investigate and improve the flow of information for a rapid response during potential future outbreaks. PHEOC in Mozambique is a stand-alone health structure, and collaboration with other health structures in surveillance is still challenging, the project of the fellow from this country focused on legal mapping to strengthen the functionality of the PHEOC in the country. Tanzania has national and regional PHEOCs, but the surveillance system at the regional level still presents major limitations that will be investigated and improved by the fellow from Tanzania. Nigeria and Ethiopia have functional and experienced PHEOCs. Through the fellowship, the fellows from these countries will promote infrastructural and workforce development projects at regional, university, and community levels. Sierra Leone has put in place several health programs based on its recent experience with Ebola and COVID-19 pandemic response. The fellow from Sierra Leone will assess and strengthen the multi-sectoral coordination and collaboration through the national PHEOC during emergency preparedness in this country. Gabon has not established a PHEOC but has established a multi-sectoral committee to respond to COVID-19 which led to a strong response to the COVID-19 pandemic. The fellow from Gabon seeks to understand the impact of this disease on other respiratory diseases like tuberculosis.

### Mentorship

To ensure the success of these projects in the different countries involved, each fellow has been paired with an experienced mentor. Mentors have continued to provide guidance to mentees on navigating the proposed project and will continue for a minimum of six months after the fellowship training program completion to supervise the individual project implementation. The mentoring program ensured an additional learning experience by leveraging diverse knowledge and experiences.

## Discussion

Africa CDC has completed its first cohort in public health emergency management. This fellowship has equipped public health emergency managers from eight different African countries reinforcing the capacity of these countries to respond to potential health emergencies. The establishment of PHEOCs in Africa started around 2012 (10). This is a command center coordinating different functions in health emergency including operations, planning, logistic and finance. These command centers have revealed a critical need of trained workforce. The US Centers for Disease Control and Prevention (CDC) has established Public Health Emergency Management (PHEM) in 2013 and built workforce suitable to run these centers (11). This PHEM Fellowship program trains international midcareer health professionals in emergency management principles through lectures, case studies, and participation in real-world. By 2021, eight years after the launch of this program, it had trained 141 health professionals in emergency management (12) and shown that graduate from the US PHEM fellowship program have served key roles in COVID-19 management in several countries and represented a key asset in their respective countries. In just one year of existence and operation, Africa CDC’s PHEM Fellowship Program has generated significant interest among African Union Member States evidenced by the feedback received at the 2023 Conference on Public Health in Africa (CPHIA 2023). In addition to in-person classes, case scenarios and study tours, the Africa CDC graduates have benefited from real-life experience in health emergency management through their participation in on-going health threats in Africa including flood response in Libya and the continental cholera outbreak response in Zimbabwe and Zambia where Fellows played vital roles alongside public health experts in PHEOC operations of the host countries. This effort is encouraged at country level for greater impact in response to diseases as seen with the Uganda public health fellowship which in its four years of operation is able to address multiple gaps in the country’s health system (13) (PMID: 3111788).

## Conclusion

The goal of emergency response in settings with limited resources is to have the greatest possible impact on public health. Preparedness plans, SOPs, and EOCs are examples of PHEM components that help respond to emergencies more quickly and effectively, resulting in a higher reduction in morbidity and mortality. The core competences of AfricaCDC PHEM are strategically tailored to address these resource gaps by equipping fellows with the desired knowledge in the context of the continents need and home country s peculiar context through the use of real time situations and peer driven solutions to identified gaps. Africa CDC PHEM Fellowship is a transformative initiative to fill the gap in workforce capacity regarding Africa’s health emergency response. There is an urgent need to train more people from different African countries. Africa CDC should implement this program with bigger cohorts to cover many African countries. This program could also be encouraged at country level in several African countries and improve workforce for epidemic response.

## Data Availability

The raw data supporting the conclusions of this article will be made available by the authors, without undue reservation.
