# Supplementary material for: The Africa Centres for Disease Control public health emergency management fellowship: insights from the inaugural cohort
Source: Front Public Health. 2025 Sep 4;13:1444354. doi: 10.3389/fpubh.2025.1444354 (PMC12443812; doi:10.3389/fpubh.2025.1444354)
Supplement: Supplementary file 1 [file Data_Sheet_1.pdf]

AFRICAN PUBLIC HEALTH EMERGENCY MANAGEMENT FELLOWSHIP

African Union

AFRICA CDC
Centres for Disease Control and Prevention
Safeguarding Africa's Health

Context

Combating the several public health threats across the African continent requires adequately trained and equipped workforce. Africa Centres for Disease Control and Prevention (Africa CDC) has called for a new public health order that includes workforce development, suitably aligned with the vision of the African Union (AU) and its desire to build capacity in public health through medium- and long-term training in identified areas of need. The commencement of the Africa CDC Public Health Emergency Management (PHEM) fellowship will carter for the dire need of skilled workforce to lead and manage emergency management programs that includes timely detection and response to emergencies when they occur.

Goal: To develop a cadre of African public health workforce who are highly skilled to coordinate and lead preparedness and response to public health emergencies in Africa.

- Provide fellows up-to-date knowledge regarding public health emergency management and Public Health Emergency Operations Centers (PHEOCs)
- Provide fellows in-depth exposure to the role of PHEOCs in preparing for and responding to various public health emergencies
- Building the skills of fellows in managing the various functions of PHEOC, and leading the response to public health emergencies
- Institutionalize a sustainable program for PHEM in the African continent

A. Fellowship Design Principles

The fellowship design considers the phenomenal nature of public health emergencies in the continent and the world's collective risks which could trigger large scale disruptions with albeit, impact the fellowship.

1. ***Fellowship Resilience***: Fellowship curriculum designed to ensure Fellows are deployable at any point of the fellowship, should there be a major public health event. Fellowship faculty will continually explore opportunities for broadening funding streams, with an institutional base funding.

2. ***Industry-savvy:*** Curriculum designed to serve the most complex emergency situations and delivered by experienced training faculty. Fellows empowered to serve at strategic, operational and tactical levels.

3. ***Scalable and adaptable Model***: As a sustainability strategy, an accountability framework is built to ensure continuous expansion and guarantee strides and lessons are easily replicable at regional, national and sub-national levels.

4. ***Leverage on existing systems:*** Fellowship goal fits into the broader goal of Africa CDC's workforce development strategy as a component of the Africa CDC New Public Health Order driven by the desire to fulfill the Africa Union's 2063 Agenda.

5. ***Continuous improvement:*** **F**ellowship will pragmatically self-evolve into an institutional flagship program, putting in place monitoring and evaluation processes that link accountability mechanisms and operational decisions to feedback from participants, AU member states and technical partners.

B. Fellowship Description

Overview

The PHEM Fellowship will target mid-career African public health professional with experience managing PHEOCs or leading emergency preparedness and response programs drawn from African Union member states. The Fellows shall be taken through a standardized training, mentorship, hands on experience, and technical assistance program leveraging on the PHEM Fellowship design of the US-CDC. During the training, fellows will receive specialized training in public health emergency management functions and operations, participate in study tours, work within Africa CDC EOC, take part in public health exercises and responses and receive guidance from global specialists in emergency management. Upon completion and return to home countries, fellows will be expected to facilitate the expansion of public health emergency management programs within their home countries and take up leadership and advisory roles in public health emergency management programs.

Fellowship Structure

The PHEM fellowship will run for 24 weeks (six months). It will include eight weeks of in-person training at the Africa CDC's headquarters in Ethiopia. During this period, Fellows will equally be matched with experienced mentors and coaches to support their continued learning experience. Fellows will then be deployed to PHEOCs across Africa including the Africa CDC PHEOC for eight weeks. The next four weeks will be spent on study tours, with each fellow getting a chance to visit one PHEOC within Africa and one outside Africa. To roundup the program, the final four weeks will be spent on didactic learning, project completion and final graduation.

Africa CDC is an equal opportunity organization.
Qualified Women are strongly encouraged to apply.

For more information related to the Fellowship, please reach us by email:
africacdceoc@africa-union.org

AFRICAN PUBLIC HEALTH EMERGENCY MANAGEMENT FELLOWSHIP

African Union

AFRICA CDC
Centres for Disease Control and Prevention
Safeguarding Africa's Health

Training Delivery

The fellowship will deploy a blended learning approach that includes online and in-person training to accomplish the goals and objectives of the fellowship. In-person sessions, webinars, keynote lectures, online self-directed study with evaluations, case studies, study tours, on-job training, and an individual mini project will all form.

Target Audience

The fellowship is designed for mid-level public health professionals with experience in managing public health emergency operations centres or leading emergency preparedness and response programs in their countries.

Eligibility Criteria

Applicants must:

a. be citizens of an African Union Member State;
b. possess a postgraduate degree in public health or related field;
c. have relevant professional experience in any field related public health including but not limited to PHEOC operations, one health, medicine, finance, logistics and supply chain health economics, health policy, animal health or environmental health;
d. be in full-time employment in any area of public health, in a public institution in Africa;
e. be able to demonstrate potentials to implement public health emergency management programs that can positively impact their countries upon return.

Selection Criteria

a. Selection shall be carried out by an independent committee comprising of experts from the African Union Commission and Africa CDC, and technical partners.
b. Selection process will be merit-based, and emphasize an equal opportunity approach to ensure the representation of underrepresented groups.

Funding

Africa CDC will provide all learning and development materials and meet all costs associated with the fellowship, including travel, monthly allowance, and insurance during the residential placements.

C. Application Process

All applicants must provide the following required information:

a. A supporting letter from the current employer to confirm employment, guaranteeing that the candidate will be allowed enough time to participate in the Fellowship program and to attend the fellowship
b. A personal statement (maximum 500 words) providing evidence of the candidate's commitment to public health emergency management in Africa detailing the following:
    - professional experience and attainment
    - Their vision of, and the future impact of their training on public health emergency management in their country and Africa
    - How they would champion public health initiatives in Africa and
    - How their personal and professional experience will enable them to fully to be excellent ambassadors of the fellowship.

C. A brief project proposal (not more than 800 words) that outlines a potential emergency management challenge they will address. The proposal should include a title, brief description of the challenge, proposed strategy for a solution and the expected outcome.
D. An updated Resume
E. Completed applications with all supporting documents should be sent to africacdceoc@africa-union.org

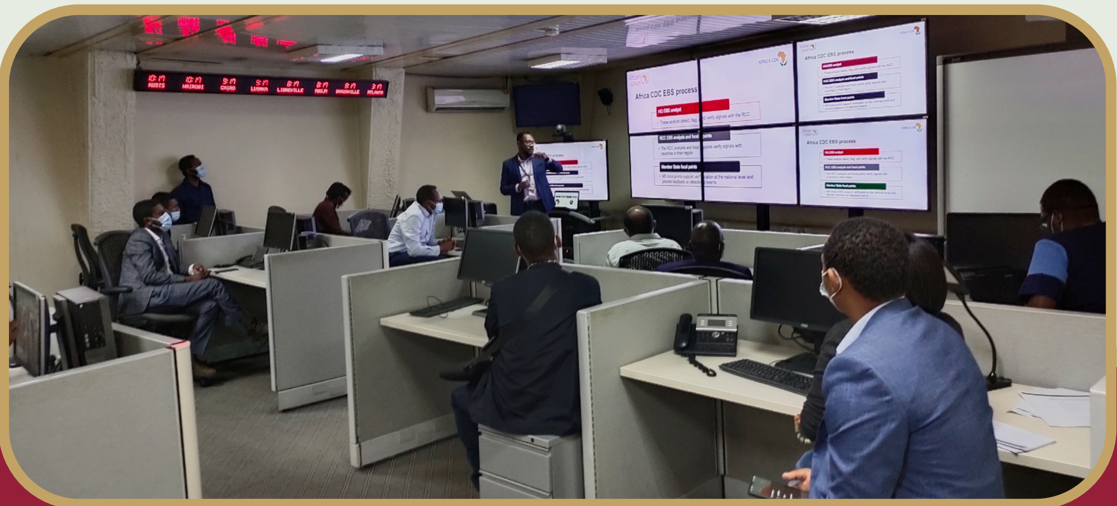

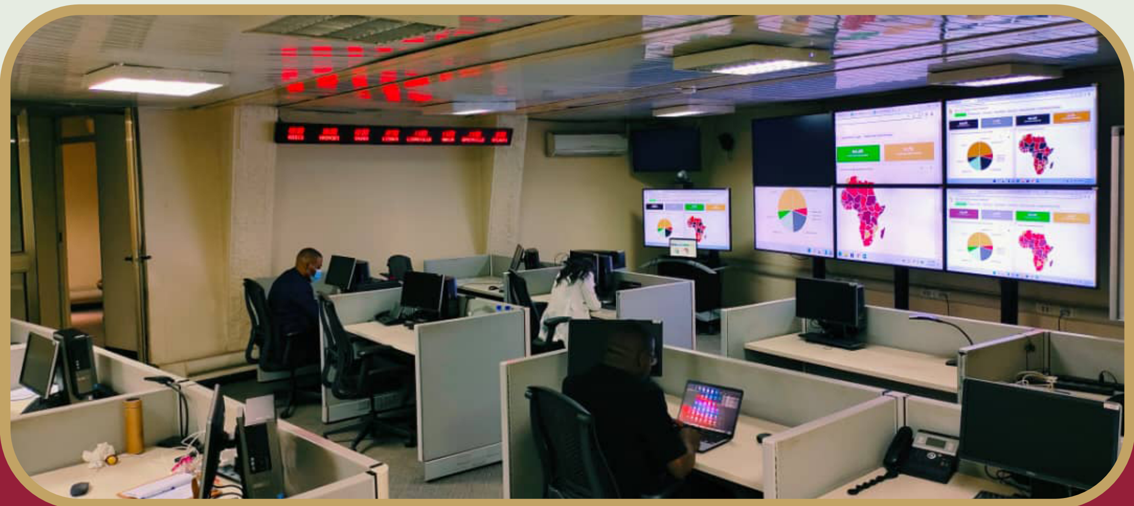

Important Dates:

► Deadline for applications: May 20, 2023

► Successful applicants will be informed by June 2023

► The fellowship will start on August 2023
